# Long-distance donor heart procurement using an innovative cold static storage system

**DOI:** 10.1177/02676591231163018

**Published:** 2023-03-11

**Authors:** Martin O Schmiady, Leszek P Bec, Mohammed Shallah, Andreas J Flammer, Paul R Vogt, Markus J Wilhelm

**Affiliations:** 1 Clinic for Cardiac Surgery, 27243 University Heart Center, Zurich, Switzerland; 2 Clinic for Cardiology, 27243 University Heart Center, Zurich, Switzerland

**Keywords:** heart transplantation, controlled cold storage, organ transportation, ischemic time, hypothermic injury

## Abstract

The global lack of donor shortage poses a major limitation for heart transplantation. New concepts with expanded donor inclusion criteria comprise extended transport distances and prolonged ischemic times with the aim of reaching a larger number of potential donors. Recent developments in cold storage solutions may allow more donor hearts with prolonged ischemic times to be use for transplantation in the future. We present our experience during a long-distance donor heart procurement with the longest reported transport distance and transport time in the current literature. This was made possible through the use of SherpaPak™, an innovative cold storage system which allows for controlled temperatures during transportation.

## Introduction

**Figure 1.**
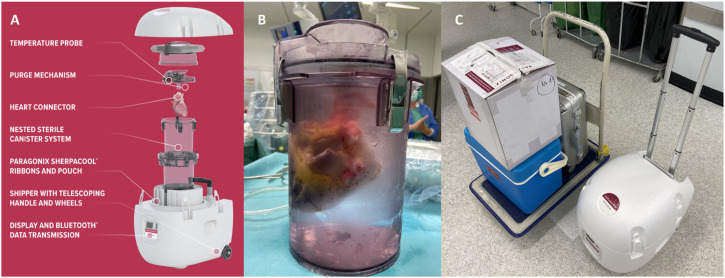
(a) The Paragonix SherpaPak™ system is a single-use, disposable cold static storage system. It consists of a sterile inner and outer canister, which are hung in a passively cooled transport unit. (b) The donor heart is fully immersed in cold cardioplegic solution in the inner canister of the system. (c) The Paragonix SherpaPak™ can be stowed compactly in two transport boxes.

Organ preservation is a fundamental component of heart transplantation. A cornerstone hereby is cold ischemia. The current standard for donor heart preservation consists of three sequential plastic bags stored in an ice box.^1^ This technique can cause freezing injuries of the donor heart as the indoor temperature is not monitored routinely.^2^ The SherpaPak™ Cardiac Transport System (CTS) (Paragonix Technologies, MA, USA) (Figure 1(a)) aims to resolve this problem by maintaining a controlled temperature between 4–8°C.^3^ After placing the sterile inner canister with the heart (Figure 1(b)) in the outer protective canister, it is passively cooled in the polystyrene transport unit by integrated cooling pads. This advanced preservation technology seems to be particularly suitable for long-distance organ transports with extended ischemic times. Thus, this new technology may help to address the problem of organ shortage by expanding the geographic range of procurement.

## Case presentation

A 55-year-old male with ischemic cardiomyopathy (LVEF 10%–15%) was listed for heart transplantation at our center. After 11 months on the waiting list a suitable brain-dead donor was identified. The donor was a 26-year-old male (162 cm, 60 Kg, BMI 22.9, BSA 1.64 m,^2^ hypoxic brain injury) with an acceptable size match to our recipient (donor-recipient weight mismatch −13%) and a good biventricular heart function (LVEF 60%–65%). The heart was procured from a center 1562 km away with a calculated flight time of 140 min. The heart was procured using standard techniques and immediately stored in the SherpaPak™.^4^ The initial starting temperature of storage was 5.4°C. Transplantation was performed through a median re-sternotomy using central cannulation and moderate hypothermic cardiopulmonary bypass. Implantation started with the left atrial anastomosis, followed by the aortic anastomosis, after which the aortic clamp was released and reperfusion initiated. During reperfusion, the remaining three anastomoses including inferior and superior vena cavae and pulmonary artery were performed on beating heart. After two defibrillations, a normal sinus rhythm was regained. Transportation time within the shipper was 225 min, and total ischemic time 294 min. The recipient cardiopulmonary bypass time and cross-clamp time was 168 and 39 min, respectively. Cardiopulmonary bypass was weaned with low-dose inotropes (3 μg/min adrenaline, 3 μg/min milrinone) and vasopressors (6 μg/min noradrenaline). Inotropic support was stopped on the first day after transplantation. Postoperative course was uneventful. Echocardiography exhibited a good biventricular function (LVEF 65%). Endomyocardial biopsies showed no signs of cellular rejection [ISHLT (1990) 0]. Patient was discharged 32 days after transplantation.

## Discussion

At present, the standard technique of heart preservation is cardiac arrest followed by static cold storage. The growing shortage of donor hearts is the main driver stimulating the development of new techniques, including normothermic ex-vivo perfusion. Current guidelines recommend a storage temperature between 5°C and 10°C.^1^ The traditional cold storage technique, consisting of three sequential plastic bags stored in an ice box, could cause hypothermic injury with impact on the graft function by uncontrolled drop in temperature or close contact with ice.^2^ Previous reports have demonstrated the effectiveness of the Paragonix SherpaPak™ in maintaining a constant storage temperature.^4,5^ In the present case organ temperature could be kept stable around 5°C even under extreme outside winter weather conditions (snow, outside temperature −10.3°C) with a high temperature variability between operation room, transport car and airplane (Table 1). As the hearts floats free in the inner container a uniform temperature distribution can be achieved (Figure 1(b)). As demonstrated in several studies, ischemic time is a major risk factor for early graft failure and rejection.^6^ Currently, the total ischemic time, as recommended by the International Society for Heart and Lung Transplantation, is up to 4 h.^7^ By maintaining donor heart temperature within the optimal range, donor hearts may tolerate longer transport and total ischemic times.

**Table 1.** Temperature courses inside the organ canister (probe temperature) and outside the shipper box (ambient temperature) during transportation.

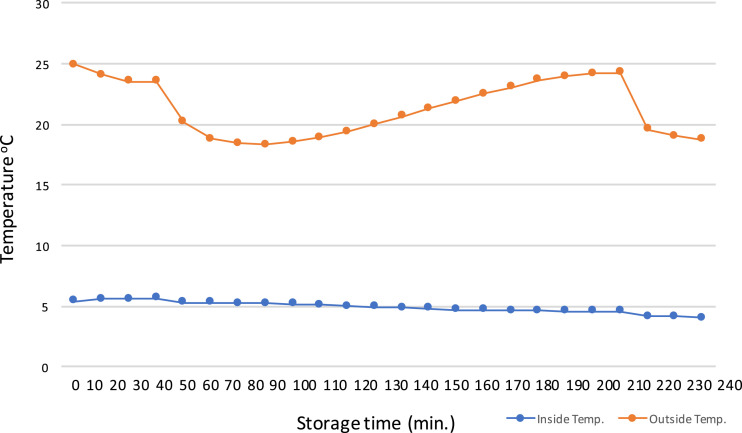

## Conclusion

The SherpaPak™ cardiac transport system provides constant organ temperatures during transportation, even in a setting with extreme weather conditions and outside temperatures. By maintaining temperatures in an optimal range organs may tolerate longer transport and ischemic times. The Paragonix SherpaPak™ is a valuable extension of the armamentarium in transplant surgery and may help to include more distant procurement centers and hereby expand the donor pool.
